# Etomidate for anesthesia induction: friends or foe in major cardiac surgery?

**DOI:** 10.1186/s13054-014-0560-7

**Published:** 2014-10-10

**Authors:** Audrey De Jong, Samir Jaber

**Affiliations:** Intensive Care Unit and Transplantation, Critical Care and Anesthesia Department (DAR B), Hôpital Saint-Éloi, CHU de Montpellier, INSERM U1046, Montpellier, France

## Abstract

Use of etomidate for anesthesia induction is still debated. In the previous issue of *Critical Care*, Heinrich and colleagues reported that etomidate for anesthesia induction had similar outcome in comparison with other drugs, in a specific population of 3,054 patients ahead of major cardiac surgery. For the authors, the similar outcomes for mortality and length of hospital stay add to the hemodynamic stability and the improved intubation conditions to support keeping etomidate in the emergency drugs armamentarium for induction of anesthesia in patients at risk of hemodynamic failure. This commentary reviews the results and implications of their study.

## Introduction

Etomidate is often considered as a two-edged sword and its use for induction of anesthesia is thus controversial. On the one hand, etomidate is used for its hemodynamic stability after anesthesia induction and also allows good intubation conditions, especially in more severely ill patients, and on the other it has negative effects on steroid synthesis. The relative importance of both the positive and negative effects for outcome of patients in both anesthesia and/or critical care is, however, a highly controversial issue.

In the previous issue of *Critical Care*, Heinrich and colleagues [1] reported in their single monocenter study that etomidate for anesthesia induction resulted in similar outcomes to other drugs in a specific population of 3,054 patients ahead of major cardiac surgery. What does this study add to what we already know?

The results of Heinrich and colleagues’ retrospective study are concordant with the recent study of Wagner and colleagues [2], a retrospective analysis of 3,127 patients receiving etomidate or propofol for induction of anesthesia; no increase was observed in severe hypotension, hospital mortality, duration of hospital stay, duration of ICU stay and duration of mechanical ventilation in the etomidate group. For the authors, this study adds evidence in support of keeping etomidate in the emergency drugs armamentarium for induction of anesthesia [3-5]. Indeed, when assessing the currently available induction agents for cardiac anesthesia, none is ideal. An ideal induction agent would be one that is easy to administer, has rapid on- and offset, and is devoid of clinically significant hemodynamic side effects. Currently, such an agent does not exist.

## Adrenal suppression

Etomidate use for anesthesia induction has been an important subject of controversy over the past years. Indeed, it has been well known that adrenal mitochondrial 11-β-hydroxylase activity is transiently inhibited by a single dose of etomidate, with consecutive adrenal suppression [6]. Whether etomidate is associated with impaired outcome is still debated and differs depending on patient categories (for example, anesthesia in the operating room, intensive care, emergency department and prehospital area). In critically ill patients suffering from sepsis, studies and meta-analyses found either an increased [7], an equal [8,9] or a decreased [3] risk of mortality after induction of anesthesia by etomidate for intubation. In major cardiac surgery, the clinical relevance of adrenal suppression after a single dose of etomidate for induction is controversial. Indeed, to our knowledge, etomidate induction has never been associated with ‘conclusive’ negative outcome data [2,10]. Again, in the current study [1], induction with etomidate was not associated with worse outcomes in comparison with other drugs.

## Systemic inflammatory response

Cardiac surgery and the initiation of cardiopulmonary bypass are stimulators of the inflammatory response. Variations in cortisol occur after major cardiac surgery and because cortisol acts synergistically with endogenous epinephrine and norepinephrine, the impairment of these stress hormones after etomidate induction is likely to contribute to an increase in vasopressor requirement. In the current study [1], the per-operative vasopressor requirement, Sequential Organ Failure Assessment (SOFA) score and inflammation blood parameters (C-reactive protein, procalcitonin and leukocytes) were not significantly different between the etomidate and the non-etomidate groups over the 5-day course observed. These results do not indicate that adverse events occur following adrenal suppression induced by etomidate. Due to the systemic inflammatory response related to cardiac surgery and the initiation of cardiopulmonary bypass, some studies provide encouraging evidence that steroids may impact on clinically important outcomes. Note that steroid utilization in the perioperative period was not described in the study of Heinrich and colleagues [1].

## Hemodynamic parameters

Post-induction hypotension has been associated with increased morbidity and mortality, both in anesthesia [11] and intensive care [4,12,13]. As an imidazole derivative, etomidate has structural similarities to α2Β-agonists, causing peripheral vasoconstriction, which contributes to the stable hemodynamic profile of this agent. Patients with cardiovascular compromise may be dependent on a high sympathetic tone to maintain their systemic vascular resistance, blood pressure, and cardiac output. For prevention of post-induction hypotension or hypertension, etomidate would appear to be more appropriate than propofol, thiopental, ketamine, or midazolam. In the current study [1], there was no difference in vasopressor requirement between the groups.

## Outcome

The similar outcomes observed in this study [1] for mortality, length of hospital stay, length of ICU stay, re-admission to ICU, duration of mechanical ventilation and SOFA score after induction with etomidate in comparison with other drugs could be the result of the opposite effects of hemodynamic stability and adrenal suppression. Moreover, better intubation conditions were found in the etomidate group, as already reported in the literature [14]. Etomidate could be associated with decreased difficult intubation incidences and complications associated with difficult intubation, even if not assessed in the study.

## Limits of the study

Some limits of the study [1] have to be pointed out. First, the drugs used in the non-etomidate group are not detailed; neither are incidences of difficult intubation and associated complications. Second, it was a single center study, which may limit the generalizability of the results. Third, all retrospective observational research, no matter how well adjusted for confounding influences, is vulnerable to residual confounding. Further randomized controlled studies are still needed to better evaluate the potential unknown confounding factors. For example, it is unclear if the patients intubated with etomidate were sicker than the patients intubated with other agents.

Nevertheless, Heinrich and colleagues [1] underline that etomidate remains one of the rare emergency drugs available for induction of anesthesia and cannot be abandoned in the setting of major cardiac surgery. Other drugs such as ketamine, thiopenthal or propofol can also be harmful in the setting of induction of anesthesia ahead of major cardiac surgery. The risk:benefit ratio of induction with etomidate should be carefully assessed before anesthetic induction of cardiac surgical patients, taking into account the associated conditions and particularly hemodynamic data. Indeed, major cardiac surgery patients differ from critically ill septic patients, and etomidate could be particularly appropriate in this situation. However, further large randomized clinical trials are needed to determine if there is any ‘optimal’ induction drug in major cardiac surgery. Analogues of etomidate, such as cyclopropyl methoxycarbonyl metomidate [15], could be of interest to retain the beneficial hemodynamic effects of etomidate with less impairment of the adrenal function.

## Conclusion

There are no convincing or consistent data from evidence-based medicine that etomidate is associated with poor outcome, especially increased mortality, particularly if adjustment is made for pre-existing severity of illness. Although some authors have suggested that, in the ICU, physicians should abandon the use of etomidate, the present study of Heinrich and colleagues [1], performed in the setting of anesthesia care, adds a new piece in the ‘etomidate puzzle’. Etomidate could be considered as an interesting and alternative drug in the setting of major cardiac surgery. Finally, it makes the decision to use or not use etomidate more and more complex: it should be used in ways that are adapted to every type of patient and reason for intubation (including type of surgery). The story of etomidate is to be continued.

## Abbreviation

SOFA: Sequential organ failure assessment
